# Labelling Assessment of Greek “Quality Label” Prepacked Cheeses as the Basis for a Branded Food Composition Database

**DOI:** 10.3390/nu14010230

**Published:** 2022-01-05

**Authors:** Evangelia Katsouri, Antonios Zampelas, Eleftherios H. Drosinos, George-John E. Nychas

**Affiliations:** 1 Hellenic Food Authority, 11526 Athens, Greece; ekatsouri@efet.gr (E.K.); azampelas@aua.gr (A.Z.); 2 Department of Food Science and Human Nutrition, School of Food and Nutritional Sciences, Agricultural University of Athens, 11855 Athens, Greece; ehd@aua.gr

**Keywords:** cheese, dairy products, food labelling, quality, PDO, PGI, GI, nutrition declaration, claims, compliance, classification, coding, data, branded food composition database

## Abstract

A labelling assessment study of Greek prepacked “quality label” cheeses was conducted with a view to provide an overview of the whole category. In total, 158 prepacked products belonging to 19 “quality label” cheeses were identified in the Greek market. Among them, Feta had the highest share followed by Kasseri, Graviera Kritis, Kefalograviera and Ladotyri Mitilinis with 81, 16, 15, 11 and 9 products found in the market, respectively. For the rest of the 14 cheeses, the share was limited, ranging from 1 to 4. All labelling indications, nutritional information, claims and other labelling data were recorded and analysed in relation to their compliance against European food law requirements. The results of the analysis showed that for only 6 of the 19 cheeses, all products fully complied with EU labelling legislation. Among the 14 mandatory labelling requirements, the lowest overall compliance was observed for allergens declaration (65%). The analysis of the nutritional data showed a remarkable variability between cheeses and products. Differences in the nutritional characteristics were more pronounced among soft, semi-hard, hard and whey cheese. The above data were entered into an archival database. Application of global harmonisation and standardisation guidelines and tools lead to the initialisation of a branded food composition database (BFCD), conceptualising a specialised database for “quality label” foods.

## 1. Introduction

Labelling laws for food and drink in Europe can be traced back to the Middle Ages (5th–14th centuries) as food marking was adopted to deliver food identity and basic properties information of the food [1]. Over time, however, under the industrialisation of food production, the domination of the retail market by packaged foods and the need for global free movement of foodstuffs, food labels evolved from simple product identity labels to complex information labels that include the food’s generic basis, nutritional composition, ingredients list, production and packaging methods, reflecting the constantly changing labelling regulatory framework, as well as the competitive global food-marketing environment. Currently, food labels constitute a multifunctional communication and marketing tool [2] delivering basic information to consumers but also intended to build an interaction between authorities, the food industry and consumers, to raise awareness on food, as well as to manage difficult public health objectives and assure the accomplishment of high marketing goals. In particular, food labels in Europe began taking their present form, with Directive 2000/13 EC [3], on purpose to enact Community rules of a general nature with detailed labelling, applicable horizontally to all foodstuffs put on the market, and are currently governed by Food Information to Consumers (FIC) Regulation (EC)1169/2011 [4].

In practice, FIC Regulation’s, labelling requirements are complemented by a number of mandatory provisions applicable to all foods, such as generic and identity information food and category name, production and packaging information, ingredients list, allergens declaration, nutritional composition either with the basic or an extended interface, date marking, etc. in order to ensure consumers’ protection. In order to help consumers suffering from allergies identify allergenic foods, allergens as ingredients have been regulated in the EU since 2003 but in view of scientific developments became an obligation under article 21 of FIC Regulation [5]. Moreover, voluntary information according to FIC or other legislative acts and policies [6,7] are also provided by the food labels. Under this context, FIC Regulation determined interpretive front-of-pack nutrition labels (FoP) schemes as a voluntary additional form of providing information in an easy-to-use way and facilitating informed consumers’ food choices [8]. Voluntary information may also include claims, specifications, schemes or marks, additional information about taste, history, origin, production methods, sustainability and quality parameters. All previous information promotes health, quality, environmental and economic goals and reduces information asymmetry between the food industry and consumers, through guiding their choices, towards quality diets and more sustainable food systems, as shown by various studies [9,10].

The EU quality labels, introduced with Regulation (EU) No 1151/2012 constitute a paradigm of such multifunctional food labels engaging with several of the previous parameters [7]. “Quality labels” include products either having a specific link to the place of manufacture and committed to satisfying certain conditions of production or products highlighting traditional aspects of production or composition, without being linked to a specific geographical area. “Quality label” products are granted either with a “geographical indication” (GI) mark, a Traditional Specialty Indication (TSG) mark or others, such as Mountain product’s, or EU’s outermost regions’ mark. They are also obliged, after passing through a specific legal procedure of approval [11], to be listed in certain quality product registers like E-Ambrosia and GIview [12]. The European Commission (EC), as part of its policy on food quality [13], has adopted the scheme of quality labels, with a view to encourage diverse agricultural production, protect product names from misuse and imitation and help consumers in their decision-making [13,14].

Geographical Indication (GIs), for foods and wine, listed in the EU geographical indications register e-Ambrosia (Official EU Database for food and agricultural products, wine, spirits and aromatised wine [10], is the most abundant category of quality labels, and comprises the following schemes.

- Protected Designation of Origin (PDO): includes agricultural products and foodstuffs (food and wine) produced, processed and prepared in a given geographical area, having the strongest link with the place of manufacturing, using recognised know-how.
- Protected Geographical Indication (PGI): includes agricultural products and foodstuffs (food and wine) closely linked to the geographical area, with one at least of the stages of production, processing or preparation taking place in the area, emphasising the relationship between the specific geographic region and the name of the product.

**Figure 1:**
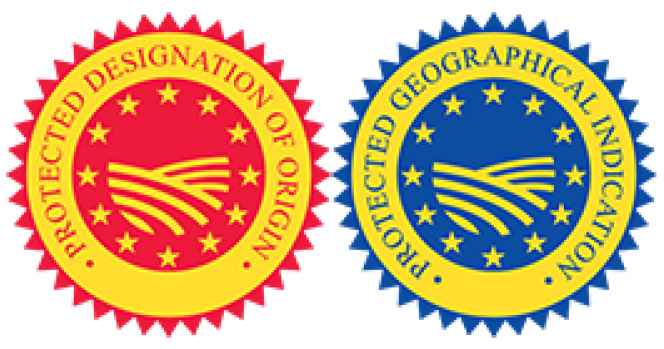
Geographical Indication (GI) marks: Protected Designation of Origin (PDO) mark and Protected Geographical Indication (PGI) mark.

Their related indication marks are shown in Figure 1:

Furthermore, these constantly evolving multifunctional food labels seem to interact in many and various ways with science, economy, consumers, academia, industry and policymakers utilising new technologies and reflecting constant scepticism about food. Branded Food Composition Databases (BFCDs) belong in the field of food labelling interaction with nutrition science [15]. BFCDs, form an evolution of food composition tables and Food Composition Databases (FCDs), adapted to processed foods with multifunctional labels. BFCDs serve the augmented need for using nutritional and other label data for diverse governmental and non-governmental activities: such as research, assessment of national health status, new product development, agricultural and food policy actions like reformulation, advertising and labelling [16,17].

**Figure 2:**
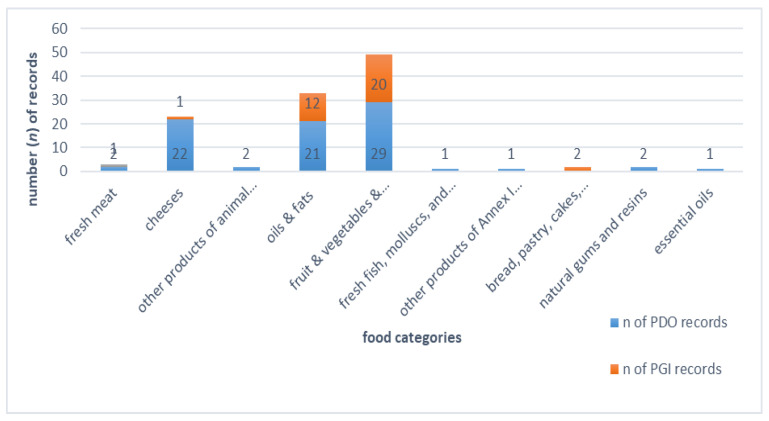
Number of records per food category, for Greece on e-Ambrosia, the EU geographical indications food register. PDO: Protected Designation of Origin, PGI: Protected Geographical Indication (PGI) mark.

Cheeses is the food category with the third higher share in quality labels of Greece (23 records of total 116 records, 19%). Fruits, vegetables and cereals category (49 records, 43%) stand in the first place and oils and fats category 3 records, 28%) in the second place. Figure 2 shows the distribution of Greek quality foods registered on EU geographical indications register e-Ambrosia [12].

Moreover, Greece is the fifth EU country in a quality label foods ranking represented by 116 food records in the European GI’s register e-Ambrosia (assessed on 20 May 2021), while Italy possesses first place with 339 food records.

Finally, cheeses comprise one of the most abundant food categories of processed food, with great variability and great importance for the domestic economy.

Based on the above, the main objective of the present study was to conduct a Labelling Assessment of prepacked Greek “quality” cheeses in order to screen their labelling status and compliance to EU legislation and explore potential problems on their labels. A second objective was to provide a nutritional syllabus for Greek cheeses utilising their nutrition declaration tables. Pilot application of a specific data structure as well as the use of standardised guidelines and tools for labelling data, during the study’s progress, allowed the creation of an archival database and the conceptualisation of its further development to a branded food composition database (BFCD) for “quality label” foods.

## 2. Materials and Methods

### 2.1. Food Category Selection and Description

The present study is focused on prepacked Greek quality cheeses. Overall 23 Greek quality label cheeses are registered in e-Ambrosia Official EU Database for food and agricultural products, wine, spirits and aromatised wine [12], including: Feta PDO (Fe), Kalathaki Limnou PDO (KL), Galotyri PDO (Ga), Katiki Domokou PDO (KD), Kopanisti PDO (Ko), Anevato PDO (An), Pichtogalo Chanion PDO (PC), Xigalo Siteias PDO (XS), Graviera Kritis PDO (GK), Graviera Naxou PDO (GN), Graviera Agrafon PDO (GA), Arseniko PDO (Ar), Kefalograviera PDO (Ke), Ladotyri Mytilinis PDO (LM), Metsovone PDO (Me), Batzos PDO (Ba), Krasotyri of Ko PGI (KK) Kasseri PDO (Ka), Sfela PDO (Sf), San Mihali PDO (SM), Formaella Arachovas Parnassou PDO (FAP), Manouri PDO (Ma), Xinomizithra Kritis PDO (XK). All cheeses belong to four different cheese categories (soft, hard, semi-hard and whey cheeses) based on their firmness according to the national Code of Foodstuffs, Beverages and Objects of Common Use (commonly referred to as the “Food Code” [18]. Abbreviations in the parenthesis above are used throughout the study instead of the full names of the cheeses. PDO mark is the dominant between Geographical Indications of Greek Quality label cheeses. Of the 23 cheeses, 22 are granted the PDO mark while only one cheese—the recently qualified Krasotyri of Ko—is granted the PGI mark.

### 2.2. Data Source (Products’ Sampling)

Original data for the analysis were sourced from all the available selected commercial prepacked “quality” cheese products’ labels and packages. Sampling was conducted from both physical retail stores and internet spots (corporate websites, online supermarkets and shops). To enhance sufficient representativeness, physical product sampling took place from stores of all major retailers of three cities in Greece (Athens, Thessaloniki, Larisa). All sampled products from physical stores were purchased and photographed through smartphones, whereas for the e-products all available information was extracted through relevant websites and saved. All photographs constituted a photo library.

The product sampling procedure took place from July 2018 until December 2020. Data from previous studies of our research team [19,20] were also used for the labelling assessment.

### 2.3. Data Collection, Data Structure Data Check and Missing Data

All information and on-pack communication of all sides for each product’s package were recorded as data in physical records (photographs and electronic files). Excel sheets including all product data and metadata were created.

**Figure 3:**
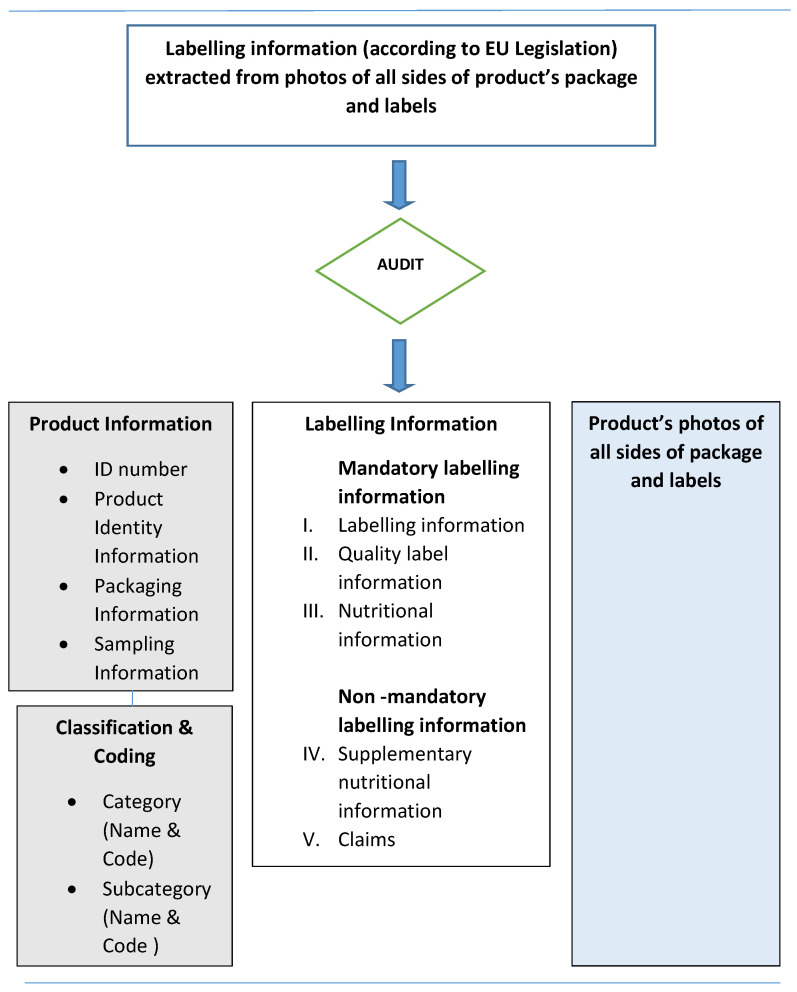
Flow-diagram presenting methodology for label data collection and structure.

During data collection, a methodology was designed in order to structure the labelling information into categories for easier recording and analysing of data in time. In this regard, data collection and data structuring were conducted considering the approach of International Network for Food and Obesity/NCD Research, Monitoring and Action Support (INFORMAS) recommendations and Food Labelling Protocol [21,22] and EuroFIR AISBL SOPs Technical Manual Version 2019–01 [23]. In order to incorporate all mandatory and voluntary information as enforced by European Legislation and existing in current food labels, an analogous procedure was formed. This procedure is shown schematically in Figure 3 and is described in detail further on.

First of all, a product single identity number (ID) was created. For each ID, the product’s respective information was reported in an excel sheet. In particular, this sheet contained the products’ sampling information (country, place, market, date of sampling, etc.), identity information, (brand name, name in own language English food name, barcode, QR code,) and packaging information (package type, packaging material, quantity-weight). In addition, the identification and description of each cheese (code and names of food category, subcategory, group, etc.) using FoodEx2, Exposure Hierarchy version Matrix 9.0 dated 26 January 2018 (downloaded 7 February 2018) [24] was attempted.

FoodEx2 is a standardised food classification and description system developed by EFSA to better describe the characteristics of foods and dietary supplements in exposure assessment studies; this system, the revised version 2, consists of flexible combinations of classifications and descriptions based on a hierarchical system for different food safety-related purposes (i.e., food consumption, chemical contaminants, pesticide residues, zoonoses and food composition). FoodEx2 system consists of 21 clearly defined food groups. Detailed food groups represent the basis of the systems; a food only fits in one group and a parent–child structure is present within the food groups. Facet descriptors, of which there are 28 in total, can be viewed as characteristics of foods from different points of view; the facets give additional information for a particular aspect of food, that is, part nature, ingredient, packaging material, production method, qualitative information, process, target consumer.

Whereupon all labelling information of each selected product was systematically arranged, per product ID number and information category. At the same time, an evaluation of compliance against EU labelling legislation mandatory requirements under the legislation was conducted. Specifically, EU Food Labelling Information System (FLIS) IT Tool for the category of cheeses [25] entailing (Reg (EU) 1169/2011(FIC) [4] and Reg (EC) 854/2005 [26] requirements, as well as European/national Legislation for GI’s [7,18] and non-mandatory requirements under Reg (EU)1924/2006 (NHCR) [6], were used. Indications required according to the EU labelling legislation and not presented on the labels (omissions or mistakes) were recorded as missing values and considered non-compliances to legislation. On the other hand, specific indications that were not obvious on corporate sites labels were considered present for the respective indication’s assessment.

In detail, all label information was firstly distinguished on mandatory and non-mandatory (voluntary) information and afterwards in further categories within the first two.

Mandatory labelling information contains:

(I) Labelling information. This category includes: all indications required in product’s label, evaluated according to Reg (EU) 1169/2011 (FIC), art.9, mandatory requirements and presented also to EU Food Labelling Information System (FLIS) IT Tool for the category of cheeses [25]. Specifically, indications required for cheeses are: food name, list of ingredients, allergens declaration, quantitative ingredient declaration QUID, net quantity, date of minimum durability, storage conditions/conditions of use, food business operator’s name and address, country of origin/place of provenance, instructions for use, nutritional declaration, lot indication, declaration of term “milk”, declaration of the animal species from the milk originates.

(II) “Quality label” information. This category includes: all data related to European “quality label” requirements according to the GI legislation, (quality mark, GI name, production establishment’s address) and national legislation mandatory requirements (category, type of milk, pasteurised or row, % min fat in dry matter and % max moisture (*w/w*), production date, packaging date, national authority’s mark with relative approval number) as well as production’s establishment’s location with production’s establishment’s approval code according to Reg (EC) 854/2005 [26].

(III) Nutritional information. This category includes: all mandatory nutritional information required and presented in the nutrition declaration table presenting food’s composition data per 100 g/mL edible portion. According to FIC Regulation, nutrition declaration table must present at minimum: energy (kJ-kcal/100 g), fat (g), saturated fat (g), carbohydrates (g), sugars (g) protein (g) and salt (g) per 100 g, in this specific order. Sometimes calculations were needed for salt estimation whenever declared as sodium, by mistake. In addition, nutrition declaration is possible to be completed by the declaration of one or more from the following components: monounsaturated, polyunsaturated, polyols, starch, fibre, vitamins and/or minerals mentioned at the Annex XIII of the FIC Regulation, components which are possible to be checked and recorded (detailed-extended nutrition declaration). Whenever information about a specific nutrient was not declared, it was recorded as missing value and non-compliance to legislation. Following the EU labelling legislation, nutrients labelled as “trace” were recorded as 0 g/100 g. Similarly, nutrient content expressed as, for example, <0.3 g, was recorded as 0.3 g.

Non-mandatory labelling information contains:

(IV) Non-mandatory supplementary nutritional information. This category includes: non-mandatory nutritional indications such as front or back of pack labelling schemes (FoPs or BoPs), information per portion (portion-size, number of portion), Reference Intake (RI) percentage on the nutrition declaration table. Thus, this category’s information is not mandatory, presence of information was recorded and evaluated. Metadata regarding FoPs, portion size were also derived and recorded.

(V) Claims, Information This category includes all claims, statements, images or any type of on-pack communication on the product. The Reg (EU) 1924/2006 (NHCR) [6] and INFORMAS protocol and taxonomy [21,22] were used for the classification of different types of claims and their presentation. According to the INFORMAS taxonomy, claims are divided into three major categories: (i) nutrition claims, (ii) health claims-compatible also to EU regulation and (iii) other claims, in which health-related claims, for example, suitable for vegans, halal, gluten-free and environment-related claims, origin and more, were included. “Organic” certification was included also in other claims. In the context of Labelling Assessment, all nutrition or/and health claims, and their conditions of use were checked according NHCR Regulation and the “Guidance on the implementation of Regulation No 1924/2006 nutrition and health claims on foods” [27] and recorded.

An Annex of the mandatory and non-mandatory labelling indications for cheeses, linked to respective Legislation, as structured data categories, is presented in Table 1 (Table S1).

The above structure provides the methodology for collecting label data, adapted to EU labels, and linked to relative EU legislation.

During data collection, a researcher specialised in auditing implementation of EU Legislation recorded in Excel sheets checked all data, initialising an archival database. Afterwards, all entries were cross-checked against the original source through the photo library.

### 2.4. Labelling Data Assessment

Structured data derived by arranging all label data from all products, according to Table 2, were considered as variables for the Labelling Assessment. In detail, we evaluated the compliance/presence of all mandatory and non-mandatory indications, respectively. The level of compliance for mandatory indications was evaluated through auditing original label data for each product and each indication against respective legislation. Absence of indications was considered non-compliance. A percentage of compliance was estimated per each indication for all products of each PDO cheese. Furthermore, an overall percentage of compliance was estimated per each indication, for all products in total.

Non-mandatory indications were evaluated in a quite similar way, by auditing the type and status of indications present on original data against respective legislation requirements, if any, and/or respective guidance documents. Regarding nutritional declaration tables, a percentage of compliance was similarly estimated for each and all cheeses. Descriptive statistics were performed for each cheese’s nutrients’ dataset, derived from all cheese products. An overview of the nutritional characteristics of each and all available PDO cheeses was provided. All statistical analysis were conducted with Excel MS Office 2010.

## 3. Results

### 3.1. Marketing Findings, Availability and Distribution of Products

**Figure 4:**
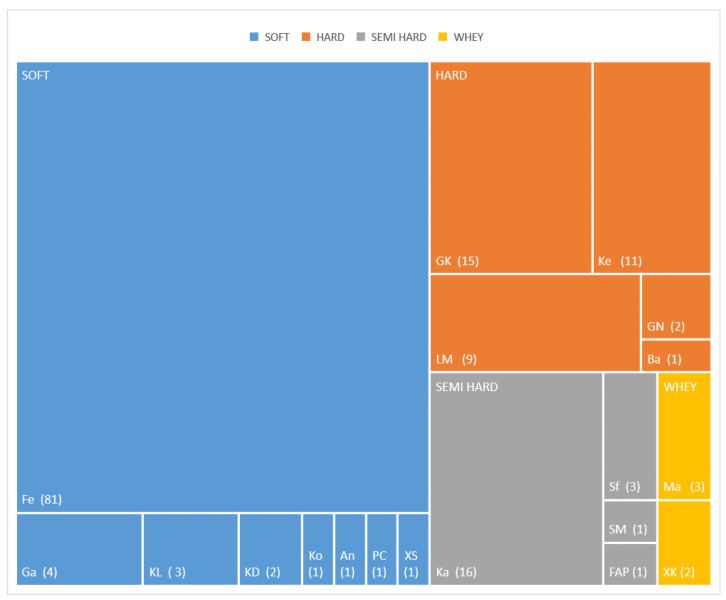
Tree map of the distribution of Greek “Quality label” cheese products identified in the retail market and grouped per cheese and firmness category. Fe: Feta PDO, KL: Kalathaki Limnou PDO, Ga: Galotyri PDO, KD: Katiki Domokou PDO, Ko: Kopanisti PDO, An: Anevato PDO, PC: Pichtogalo Chanion PDO, XS: Xigalo Siteias PDO, GK: Graviera Kritis PDO, GN: Graviera Naxou PDO, Ke: Kefalograviera PDO, LM: Ladotyri Mytilinis PDO, Ba: Batzos PDO, Ka: Kasseri PDO, Sf: Sfela PDO, SM: San Mihali PDO, FAP: Formaella Arachovas Parnassou PDO, Ma: Manouri PDO, XK: Xinomizithra Kritis PDO.

In total 158 “quality label” prepacked cheese products were identified in the Greek market. All products belonged in 19 of the 23 cheese records of the Greek “quality cheeses” list [28]. In detail, the number of products collected per cheese were: Feta PDO (*n* = 81), Kalathaki Limnou PDO *(n* = 3), Galotyri PDO (*n* = 4), Katiki Domokou PDO (*n* = 2), Kopanisti PDO (*n* = 1), Anevato PDO (*n* = 1), Pichtogalo Chanion PDO (*n* = 1), Xigalo Siteias PDO (*n* = 1), Graviera Kritis PDO (*n* = 15), Graviera Naxou PDO (*n* = 2), Kefalograviera PDO (*n* = 11), Ladotyri Mytilinis PDO (*n* = 8), Batzos PDO (*n* = 1), Kasseri PDO (*n* = 16), Sfela PDO (*n* = 3), San Mihali PDO (*n* = 1), Formaella Arachovas Parnassou PDO (*n* = 1), Manouri PDO (*n* = 3), Xinomizithra Kritis PDO (*n* = 2). No products of Graviera Agrafon PDO, Arseniko PDO, Krasotyri of Ko PGI, Metsovone PDO were found to be marketed as prepacked. The product distribution among the different cheeses available in the Greek retail market is presented in Figure 4.

As shown in the above distribution by the comparative number of products that were found on the market, Feta cheese possesses the greatest market share among Greek quality cheeses (81 products found in the market). Kasseri (16 products) comes second while Graviera Kritis (15 products), Kefalograviera (11 products) and Ladotyri Mytilinis (9 products), following in descending order. The rest of the cheeses are rarely found in the market, 11 of the 23 (47.8%) having none or just one representative.

### 3.2. Labelling Assessment of Greek Prepacked “Quality Label” Cheeses

A labelling assessment was conducted for branded Greek prepacked “quality label” cheeses, attempting an overall mapping of the category for the first time. The specific results of the assessment are presented in the following sections.

#### 3.2.1. Assessment of Mandatory Labelling Information

The level of compliance for each mandatory indication according to EU Food Labelling Information System (FLIS) IT Tool for cheeses was assessed for all 158 products identified in the Greek market. In particular, the following indications, also described in paragraph 2.3 (I), were evaluated. At first, FIC Regulation’s, art.9, (11 indications): 1. food name, 2. ingredients list, 3. allergens declaration, 4. quantitative ingredient declaration QUID, 5. net quantity, 6. date of minimum durability, 7. storage conditions/conditions of use, 8.food business operator’s name and address, 9. country of origin or place of provenance, 10. instructions for use, 11. nutritional declaration table. Next, particular indications according to specific legal provisions (three indications): lot number, use of term “milk” and the animal species from which the milk originates. In terms of the present assessment, ingredients list indication, even though it is not always mandatory for cheeses, was considered and evaluated as such.

The results on the compliance for each mandatory indication, according to FIC Regulation, art. 9, for each cheese separately and for all cheeses (overall) based on the total 158 products identified in the Greek market are presented in Table 2.

The results based on Table 2 showed that the majority of mandatory labelling requirement indications according to FIC Regulation are provided correctly to consumers (100% compliance). However, specific omissions and/or non-compliances were observed for certain cheeses and indications.

In particular, among the 14 mandatory labelling indications, the lowest overall compliance was observed on allergens declaration (65%) followed by ingredients list (79%), QUID (90%) and nutritional declaration (92%). For allergen declaration, 100% compliance was found for only six cheeses, while in five, it was totally missing and in the rest of the eight cheeses, it was partly missing. Ingredients list and QUID were found to be fully present (100% compliance) only in 8 and 12 cheeses, respectively, while for the rest of the cheeses, the above mandatory indications were totally or partly missing. The absence of ingredients lists seemed to relate to the allergen declaration omission. Thus, quite often when the ingredients list was absent, allergens were also not declared. Similarly, the nutrition declaration table was absent in various percentages in six cheese categories. Minor nutrition declaration non-compliances were observed for the most abundant cheeses (Fe, KL, GK, Ke, LM, Ka) as expected, due to the multitude of the products with percentages of compliance ranging from 67–96%. The above non-compliances were related mainly to the nutrition declaration table plenitude and the correct sequence of nutrients. The rest of the mandatory indications are presented in Table 2, for the majority of products, in general, they were found to be fully provided. In detail, food name, net quantity, date of minimum durability, storage conditions/conditions of use, food business operator’s name- address and instructions for use were present in the products’ labels with very high percentages of compliance ranging from 95–100%.

From the cheeses point of view (based again on Table 2): six cheeses (PC, Ba, SM, FAP, Ma, XK) were found in full compliance (100% compliance in all indications), and six cheeses (Fe, KL, GK, GN, Ke, LM) presented non-compliances on up to three indications. Moreover, five cheeses (KD, Ko, An, XS, GN) were found to totally lack allergen declarations (0% compliance).

Regarding specific label information extracted as metadata, from the labels and not presented in Table 2, such as durability time, way of declaration of durability time, milk species from which the cheeses originate, they were also recorded and assessed. Durability time of products was found to vary between and within cheese categories. Thus, although soft cheeses display an average durability time of 21 months, max durability time in soft creamy cheeses like An and PC barely approached 1–2 months, while F found reaching 24 months. Moreover, hard and semi-hard cheeses display average durability times of 11–14 months, while whey cheeses had up to 9. Regarding the way of declaration of durability times, they were found to be expressed both as “best before” and “use by date” in all cheese categories, while there were also many products in total, declaring durability times with expressions such as “expiry or expiration date“, which is not compliant. The milk species from which Greek “quality cheeses” originate are mainly sheep and goat’s milk, while cow’s milk is used only in the production of Graviera Naxou, Kefalograviera and Kopanisti.

Regarding mandatory indications according to “quality label” legislation, non-compliances were observed infrequently and mainly in small-scale production firms. Almost all commercialised cheese products were found to bear the PDO mark. The observed scarce omissions and non-compliances were found to be mainly related to packaging date and “quality label” packaging identification number, which was often found to be confused with the lot number. Quite often though, the production establishment’s approval code number was found to be incorrectly expressed.

#### 3.2.2. Assessment of Non-Mandatory Labelling Information

Non-mandatory labelling information including voluntary supplementary nutritional information (FoPs, per portion information, % RI) and claims was also assessed.

In 9 of 19 cheeses, FoP schemes were found to be provided at a 29% overall percentage. The types of FoP schemes observed, were: of only Energy or Energy+ type based on the Guideline Daily Amount (GDA) system [29] were not always placed on the front side of the package. Furthermore, in 5 and 8 of 19 cheeses, per portion information (portion size, number of portions) and % RI information were provided, in percentages of 35% and 31% average, respectively. The portion sizes were declared only in a few packages and varied between 20–50 g in all cheeses.

Regarding claim data findings in relation to NHCR Regulation provisions, nutrition and health claims were rarely displayed on Greek “quality label” cheeses. In detail, only one specific comparative nutrition claim was observed in 7.4% of Feta products (5.7% overall). The claim that was recorded in the above cases was the comparative nutrition claim “40% less salt” which was always in full compliance with the claim’s conditions of use according to NHCR Regulation’s requirements. Sometimes, the nutrition claim “low salt” was also observed in Feta products and the statement: “only 13% fat” in Katiki Domokou and Galotyri products, always non-compliant to legislation’s requirements. No claim regarding calcium content, such as “source of calcium” or “rich in calcium” was recorded, even though calcium concentrations of the products could probably support these nutrition claims. No other nutrition or health claims were observed.

Other claims or symbols/marks checked and reported, were mainly claims of “origin” and “organic” type. “Origin” claims were displayed either with a nationally regulated heart-shaped Greek flag or with a simple Greek flag and/or with the statement: “Greek product”. As far as organic claim concerns: it was displayed either with the statements: “organic”, “certified organic” or “bio”, always accompanied by the European symbol for organic certification. The “Organic” claim was observed in five cheese categories and a 6.3% overall percentage. Regarding the “no preservatives” statement, it was identified in quite a few cases. No sustainability, environmental, “natural or health-related” type claims were observed, while at the same time, the recycling mark was very often present.

#### 3.2.3. Assessment of Nutritional Information Data in Relation to FIC Regulation Provisions

Regarding nutritional information data, the nutrition declaration table was displayed in cheeses at a 92% overall percentage. Furthermore, absence of specific nutrients or differences from the standard sequence of nutrients on the nutrition declaration table in terms of the current evaluation constituted non-compliances to FIC. Only an l8.2% overall percentage of the products, (mainly products of Feta), comprised micronutrients concentration (only calcium), in their tables. Fibre, a conditionally declared nutrient according to the FIC, was always assigned 0, either declared so or not, in the cheeses’ tables

The analysis of the nutritional data of quality cheeses, showed—as expected—remarkable variability between the PDO cheeses and products, in all critical macronutrients. Descriptive statistics for nutrients’ contents, conducted for each cheese product and total summarised results are presented in Table 3.

With respect to the above statistics, various comments can be made. For example, in soft-brined Feta cheeses, salt ranges from 0–5 g/100 g, in hard aged Graviera Kritis cheeses from 0.78–2 g/100 g while in soft creamy Katiki Domokou raises up to 1 g/100 g). Regarding saturated fat, whey Manouri displays the greater concentration, among all quality cheeses, ranging from29–34.8 g/100 g due to its production technology (addition of whipping cream during production procedure). At the same time, between whey “mizithra” cheeses a great variability was observed in total fat, saturated fat and protein content between Xinomizithra Kritis and Manouri.

As far as protein is concerned, Manouri had the lowest concentration of 6/100 g, and we found protein concentrations up to 30.6/100 g in Graviera Kritis. Finally, regarding calcium, quite high concentrations were observed wherever calcium was declared (up to 500 mg/100 g on Feta, 783 mg/100 g on Kefalograviera, 942 mg/100 g on Ladotyri Mytilinis), a fact that is definitely supported by other studies [19,20].

### 3.3. Initialising an Archival Database and Conceptualising a Branded Food Composition Database for “Quality Label” Foods

The implementation of the previously described procedure of arranging label data in order to conduct a comprehensive labelling assessment for Greek “quality cheeses’” (Table 1), led us to the initialisation of a database. Label data of original products were entered into an archival database, considering existing harmonisation and standardisation guidelines and tools (INFORMAS recommendations and Food Labelling Protocol [21,22], EuroFIR AISBL [23] and FoodEx2 [24].

In the absence of a standard methodology for the development of a database, the previously described procedure, not only provided a methodology for labelling data collection but furthermore formed the basis for the conceptualisation of a branded food composition database (BFCD) for “quality label” foods.

**Figure 5:**
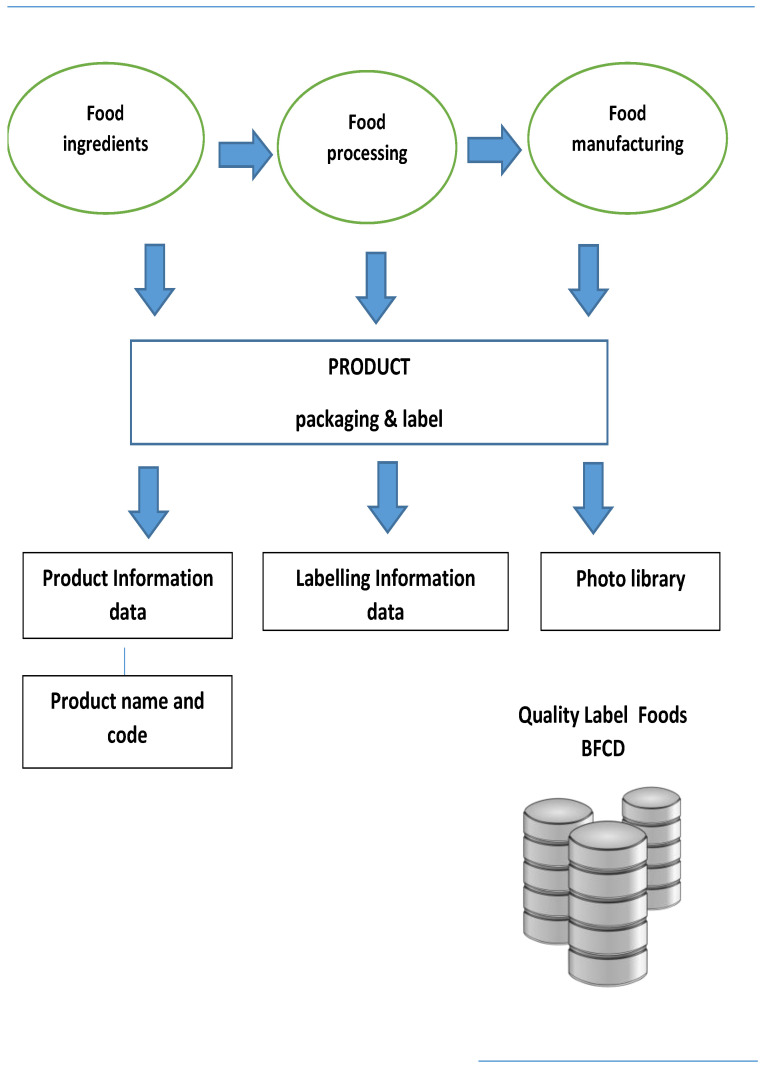
Flow-diagram presenting methodology for and the design and development of a branded food composition database (BFCD) for “quality label” foods.

A graphical representation of the conceived methodology for the potential development of a BFCD is presented in Figure 5.

Regarding the current status of the concept, a total of 158 products were entered into the first version of an archival database, intended for further development with other “quality label” foods. Reported data entered until February 2021.

In reference to classification and description according to FoodEx2, the “Milk and Dairy products” (A02LR) food category and the “Cheeses” (A02QE) subcategory were matched. All the above-described quality label cheeses were found to belong in three of the six subgroups of the above subcategory, and specifically in fresh uncured cheeses (A02QF), brined cheeses (A02RA) and in ripened cheeses (A02RG) subgroups. In total, the following 13 descriptors of the FoodEx2 system were identified: Cheese (A02QE), fresh uncured cheese (A02QF), miscellaneous fresh uncured cheeses (A04NV), cheese mizithra (A02QV), brined cheese (A02RA), feta type and similar soft brined cheese (A02RB), feta (A02RB), firm brined cheese (A02RE), firm ripened cheeses (A02ST), firm semi-hard cheeses (A02SV), kasseri (A02VG), hard cheese (A02YE), aged graviera (A02YF).

During the classification and coding procedure, more than half of the Greek PDO cheeses could not be accurately described with existing descriptors and for those, many cheeses were assigned with the wider category code. A limited number of FoodEx2 system descriptors regarding cheeses was observed and the article supports a possible expansion.

## 4. Discussion

The marketing findings presented in the first part of the study’s results, in relation to the availability of pre-packed Greek “quality label” cheeses indicated significant problems in their marketing potential, inside the domestic market and definitely abroad. Indeed, 4 out of total 23 “quality label” Greek cheeses were not found at all in the retail market of the three major cities that sampling was carried out in. In addition, 132 (84%) out of total 158 products identified in the market represent 5 cheeses (Feta, Graviera Kritis, Kasseri, Kefalograviera, Ladotyri Mytilinis) and only 26 (16%) of total 158 products represent the rest of the 14 cheeses. These findings prove that many Greek “quality cheeses” do not reach easily make way to the market, a fact that also impacts their state of awareness. This is definitely indicative of the “quality labels” market footprint in regard to their identity and characteristics. Undoubtedly all the above findings are in line with the recent “Evaluation support study on geographical indications and traditional specialties guaranteed protected in the EU: final report” [30], which confirms that “quality label” products, are facing a lack of awareness. Moreover, they also confirm specific conclusions from a recently published review study, on the GI’s market and economic issues [31]. Possible reasons for the limited market representation of “quality cheeses” in Greece could be poor state marketing support and missing marketing strategies also reported in other European countries [32] or other indigenous reasons. Indicatively, we can mention the limited production rate, which is linked to the nature of the products (seasonality of production, small scale production firms, local production and sales) as well as the concession of livestock-farming and the reduction in the availability of raw milk, but more research has to be conducted on these issues.

The labelling assessment of prepacked Greek “quality cheeses” presented in the second part of the study’s results, depicted their labelling status and compliance to EU legislation, explored problems on their labels and provided a complete overview of their nutritional characteristics for the first time. Mandatory and non-mandatory labelling information of 158 products belonging to 19 cheeses was identified and assessed. The results of the assessment showed a certain pattern of omissions and non-compliances regarding mandatory requirements. Non-compliances in allergen declaration, ingredient list, QUID and nutrition declaration indications were most frequently observed and mainly in brands of small size and scale firms. As far as non-mandatory information is concerned, results showed that claims, innovative tools and on-pack communication information and schemes (such as FoPLs) had limited representation on Greek “quality label” cheeses, although many studies have shown that they can help consumers in better understanding nutritional information of food [33,34]. Sustainability marks were also totally absent. Nutritional declaration tables served for conducting a comprehensive statistical analysis of the nutritional characteristics of all Greek available “quality label” cheeses, which were presented comparatively per cheese and cheese category. The above assessment, results and information constitute the first study on mapping the labelling status and nutritional characteristics of all “quality label” cheeses in Greece and one of the scarce studies found on labelling compliance assessment against regulated information that should be provided to consumers. The study’s results provide important information for Authorities and FBOs, in order to facilitate labelling requirement monitoring procedures and improve cheeses’ labels.

The creation of an archival database and the conceptualisation of a branded food composition database (BFCD), presented in the third part of the study’s results, was conducted with the view to better the possible depiction of “quality label” cheeses. All steps were designed and carried out using standardised guidelines and tools [21,22,23,24] while global trends that have been adopted by national BFCDs (such as OQALI [35], USDA BFCD [16], UK BFCD [36], NUBEL [37] and HelTH [17] as well as by specific specialised databases [38] were followed. Considering also that global harmonisation and standardisation tools and standardised compilation procedures of data support FAIR (Findable, Accessible, Interoperable, Reusable) data processing, the whole project stays definitely in line with the FAIR data principles adapted to the agrifood sector [39]. The idea of a “quality label” food database follows, in a way, the previous specialised databases of traditional and ethnic foods and bioactive compounds [40]. Specialised databases are databases that can capture more detail (e.g., specific descriptions of the foods components identification, values, and measures of variability) and also serve for other uses [41]. The prospect of the creation of a specialised branded food composition database deserves scientific attention, considering the lack of centralised data collection about “quality label” products on the EU level [31]. Except for the official registration databases [12], only specific initiatives for Geographical Indication (GI) products’ data collection were found in EU countries with a strong GI industry, (e.g., Qualivita in Italy) [31,42]. A specialised “quality label” BFCD may contribute to better identification of all available “quality” products, considering also that many “quality foods” have not been described yet in terms of classification, as also shown in the present study. Such a database may additionally constitute a comprehensive tool for stakeholders (industry, research and policymakers) supporting them in new product development, product reformulation, food promotion, monitoring, keeping track of changes using other new technologies (e.g., immutable ledgers such as blockchain approach, etc.) both from the nutritional point of view and as a key tool for public health [43,44,45]. Better identification of existing problems related to the “quality labels” could facilitate both producers and policymakers in improving the marketing strategies of the labels and in more effectively managing the benefits arising from the certification [32]. In the future, typical dairy and meat products will only be able to maintain and develop their markets if they are capable enough of holding their commercial ground and adapting to the market’s needs and demands without losing their specificity, originality and authenticity [46]. In addition, the rapidly changing food markets and new nutritional and health interests create both needs and gaps in existing food composition databases and the availability of branded food databases provides new opportunities and challenges [47].

## Figures and Tables

**Table 1:** Annex of labelling indications–data categories’ structure, used for label data collection accompanied with relative EU legislation.

Annex			
	Label	Labelling Indication/Data	EU Legislation
Mandatory information	I. Labelling information	Food name	Reg. 1169/2011
		Ingredient list	Reg. 1169/2011
		Ingredients (extensively)	Reg. 1169/2011
		Allergen declaration	Reg. 1169/2011
		Quantitative ingredient declaration (QUID)	Reg. 1169/2011
		QUID list	Reg. 1169/2011
		Net quantity	Reg. 1169/2011
		Date of minimum durability	Reg. 1169/2011
		Durability date type	Reg. 1169/2011
		Durability date time	Reg. 1169/2011
		Storage conditions/conditions of use	Reg. 1169/2011
		Food business operator’s name and address	Reg. 1169/2011
		Country of origin or place of provenance	Reg. 1169/2011
		Instructions for use	Reg. 1169/2011
		Nutrition declaration table presence	Reg. 1169/2011
		Lot indication	Reg. 1308/2013
		Use of term “milk”	Reg. 1308/2013
		Animal species from which the milk originates	Reg. 1308/2013
	II. Quality label information	Type of milk	National Code, art.83, general requirements
		% min fat on dry matter	National Code, art.83, general requirements
		% max humidity *w/w*	National Code, art.83, general requirements
		Production date	National Code, art.83, general requirements
		Packaging date	National Code, art.83, general requirements
		Packaging identification number	National Code, art.83, general requirements
		Quality label mark	National Code, art.83, Traditional cheeses
		Food name as registered	National Code, art.83, Traditional cheeses
		Production establishment’s address	National Code, art.83, Traditional cheeses
		National authority’s approval number and mark	National Code, art.83, Traditional cheeses
		Production establishment’s approval code number	Reg. 854/2004
	III. Nutritional information	Energy/Energy unit Protein/Protein unit Total fat/Total fat unit Saturated fat/Saturated fat unit Trans fat/Trans fat unit Carbohydrates/Carbohydrates unit Sugar/Sugar unit Fibre/Fibre unit Salt/Salt unit (insert extra row for each extra nutrient if any)	Reg. 1169/2011
		Nutrition declaration mandatory particulars	Reg. 1169/2011
Non-mandatory information	IV. Nutritional supplementary information	Portion particulars	Reg. 1169/2011
		Portion size	Reg. 1169/2011
		RI’s particulars	Reg. 1169/2011
		Front of Pack Label schemes (FoPs)	Reg. 1169/2011
		Type of FoP	Reg. 1169/2011
	V. Claims information	Type of claim for each claim	Reg. 1924/2006 -INFORMAS taxonomy
		Wording of claim for each claim	Reg. 1924/2006
		Placement of claim for each claim	Reg. 1924/2006
		Format of claim for each claim	
		Total number of claims for each product	
		Nutrition claims’ total number	
		Health claims’ total number	
		Other claims’ total number	
		Other marks-symbols type	

**Table 2:** Percentage (%) of compliance for eacmandatory labelling indication according to FIC Regulation’s, art. 9, for 158 pre-packed cheese products belonging to 19 cheeses identified in the Greek market.

Cheese Category	Soft								Hard					Semi hard				Whey		
Cheese	Fe	KL	Ga	KD	Ko	An	PC	XS	GK	GN	Ke	LM	Ba	Ka	Sf	SM	FAP	Ma	XK	Overall
Count of products	81	3	4	2	1	1	1	1	15	2	11	9	1	16	3	1	1	3	2	158
Food name	95	100	100	100	100	100	100	100	100	100	100	100	100	100	100	100	100	100	100	97
Ingredients list	73	100	100	100	100	100	100	0	93	50	82	89	100	75	67	100	100	100	100	79
Allergens declaration	65	67	25	0	0	0	100	0	80	0	64	56	100	81	33	100	100	100	100	65
Quantitative ingredient declaration (QUID)	100	67	100	100	100	100	100	100	60	50	73	89	100	75	100	100	100	100	100	90
Net quantity	100	100	100	100	100	100	100	100	100	100	100	100	100	100	100	100	100	100	100	100
Date of minimum durability	100	100	100	100	100	100	100	100	80	100	100	100	100	100	100	100	100	100	100	98
Storage conditions/conditions of use	99	100	100	100	100	100	100	100	100	100	100	100	100	100	100	100	100	100	100	99
Food business operator’s name and address	99	100	100	100	100	100	100	100	100	100	100	100	100	100	100	100	100	100	100	99
Country of origin or place of provenance	100	100	100	100	100	100	100	100	100	100	100	100	100	100	100	100	100	100	100	100
Instructions for use	100	100	100	100	100	100	100	100	100	100	100	100	100	100	100	100	100	100	100	100
Nutrition declaration table	96	67	100	100	100	100	100	100	80	100	82	89	100	88	100	100	100	100	100	92

Colours assigned for overall compliance: 0–70% red, 70–90% orange, 90–100% yellow, 100% green. Fe: Feta PDO, KL: Kalathaki Limnou PDO, Ga: Galotyri PDO, KD: Katiki Domokou PDO, Ko: Kopanisti PDO, An: Anevato PDO, PC: Pichtogalo Chanion PDO, XS: Xigalo Siteias PDO, GK: Graviera Kritis PDO, GN: Graviera Naxou PDO, Ke: Kefalograviera PDO, LM: Ladotyri Mytilinis PDO, Ba: Batzos PDO, Ka: Kasseri PDO, Sf: Sfela PDO, SM: San Mihali PDO, FAP: Formaella Ara-chovas Parnassou PDO, Ma: Manouri PDO, XK: Xinomizithra Kritis PDO.

**Table 3:** Nutritional composition of Greek prepacked “quality label” cheeses, according to their labelling nutrition declaration tables.

Cheese Category	Cheese	Count of Products	Energy (Kcal/100 g)	Total Fat (g/100 g)	Saturated Fat (g/100 g)	Carbohydates (g/100 g)	Sugar (g/100 g)	Protein (g/100 g)	Salt (g/100 g)	Calcium (mg/100 g)
Soft	Fe	81	280.5 ± 20.3	23.4 ± 1.6	15.9 ± 1.4	0.9 ± 0.8	0.5 ± 0.6	16.6 ± 1.1	2.4 ± 0.7	410.0 ± 109.5
	KL	3	276.0 ± 0.0	23.0 ± 0.0	16.5 ± 0.0	1.8 ± 0.0	0.0	15.4 ± 0.0	3.0 ± 0.0	
	Ga	4	155.0 ± 10.9	12.1 ± 1.7	7.2 ± 1.2	2.0 ± 1.9	2.0 ± 2.4	10.8 ± 2.3	1.6 ± 0.0	
	KD	2	166.0 ± 4.2	13.0 ± 0.0	8.0 ± 0.0	2.0 ± 1.4	3.0	10.5 ± 0.7	1.0 ± 1.0	
	Ko	1	304.0	24.0		0.5		22.0		
	An	1	210.0	17.5	12.0	0.7	0.7	12.5	2.5	
	PC	1	127.0	6.7	4.8	4.5	4.2	12.1	0.7	
	XS	1	163.0	11.9	8.5	4.3	1.9	9.4	1.2	
Hard	GK	15	399.2 ± 31.8	30.8 ± 3.9	21.5 ± 2.8	1.9 ± 1.0	0.9 ± 0.8	26.3 ± 2.3	1.6 ± 0.6	
	GN	2	419 ± 55.2	31.3 ± 0.4	20.1 ± 0.2	1.6 ± 1.6	1.5 ± 1.7	24.7 ± 1.8	2.1 ± 0.0	
	Ke	11	378.7 ± 15.5	30.7 ± 1.3	20.7 ± 1.6	0.5 ± 0.9	0.1 ± 0.1	25.3 ± 1.6	2.6 ± 0.4	783.0
	LM	9	370.8 ± 24.8	30.3 ± 3.2	19.2 ± 1.5	0.4 ± 0.3	0.0 ± 0.0	24.2 ± 2.8	2.9 ± 1.3	942.0
	Ba	1	344.0	25.0	17.3	0.7	0.7	29.0	2.5	
Semi Hard	Ka	16	345.9 ± 23.8	27.9 ± 2.8	18.7 ± 1.8	0.7 ± 0.7	0.2 ± 0.2	24.4 ± 1.4	2.1 ± 0.3	
	Sf	3	327.6 ± 12.6	27.9 ± 2.2	19.8 ± 0.7	0.2 ± 3.4	0.2 ± 3.4	19.9 ± 4.9	3.4 ± 0.6	
	SM	1	334.0	23.0	16.0	4.8	0.2	27.0	2.5	
	FAP	1	340.0	25.0	17.4	0.7	0.1	21.0	2.5	
Whey	Ma	3	487.0 ± 62.2	46.9 ± 4.6	32.8 ± 3.3	1.5 ± 0.6	1.2 ± 0.6	7.8 ± 2.0	1.4 ± 0.6	
	XK	2	280.5 ± 5.0	21.5 ± 0.7	15.0 ± 0.0	3.0 ± 0.0	1.8 ±1.8	17.5 ± 0.7	1.7 ± 1.2	

Fe: Feta PDO, KL: Kalathaki Limnou PDO, Ga: Galotyri PDO, KD: Katiki Domokou PDO, Ko: Ko-panisti PDO, An: Anevato PDO, PC: Pichtogalo Chanion PDO, XS: Xigalo Siteias PDO, GK: Gra-viera Kritis PDO, GN: Graviera Naxou PDO, Ke: Kefalograviera PDO, LM: Ladotyri Mytilinis PDO, Ba: Batzos PDO, Ka: Kasseri PDO, Sf: Sfela PDO, SM: San Mihali PDO, FAP: Formaella Ara-chovas Parnassou PDO, Ma: Manouri PDO, XK: Xinomizithra Kritis PDO.

## Data Availability

Data used and presented in the study are openly available in the products labels. Analyzed dataset was archived under the development of the branded food composition database (BFCD).

## Supplementary Materials

The following are available online at https://www.mdpi.com/article/10.3390/nu14010230/s1, Table S1. Annex of labelling indications–data categories’ structure, used for label data collection accompanied with relative EU legislation.
