# Supplementary material for: Labelling Assessment of Greek “Quality Label” Prepacked Cheeses as the Basis for a Branded Food Composition Database
Source: Nutrients. 2022 Jan 5;14(1):230. doi: 10.3390/nu14010230 (PMC8747277; doi:10.3390/nu14010230)
Supplement: Supplementary file 1 [file nutrients-14-00230-s001.zip › nutrients-1500328-supplementary.pdf]

Table S1. Annex of labelling indications–data categories' structure, used for label data collection accompanied with relative EU legislation.

ANNEX				
Label		Labelling Indication/ data	Description of data	EU legislation/ other
Mandatory information	I. Labelling information	Food name	food name	Reg. 1169/2011
		Ingredients list	presence (0=no, 1=yes)	Reg. 1169/2011
		Ingredients	list of ingredients in English, additives reporting	Reg. 1169/2011
		Allergens declaration	presence (0=no, 1=yes)	Reg. 1169/2011
		Quantitative ingredient declaration (QUID)	presence (0=no, 1=yes)	Reg. 1169/2011
		QUIDlist	list of ingredients in English, separated by semicolon	Reg. 1169/2011
		Net quantity	presence (0=no, 1=yes)	Reg. 1169/2011
		Date of minimum durability	presence (0=no, 1=yes)	Reg. 1169/2011
		Durability date type	1. best before date<br>2. use by date<br>3. other	Reg. 1169/2011
		Durability date time	durability time in years/months/days	Reg. 1169/2011
		Storage conditions / conditions of use	presence (0=no, 1=yes)	Reg. 1169/2011
		Food business operator's name and address	presence (0=no, 1=yes)	Reg. 1169/2011
		Country of origin or place of provenance	presence (0=no, 1=yes)	Reg. 1169/2011
		Instructions for use	presence (0=no, 1=yes)	Reg. 1169/2011
		Nutrition declaration table presence	presence (0=no, 1=yes)	Reg. 1169/2011
		Lot indication	presence (0=no, 1=yes)	Reg. 1308/2013
		Use of term "milk"	presence (0=no, 1=yes)	Reg. 1308/2013
		Animal species from the milk originates	1. bovine<br>2. ship<br>3. goat<br>4. other	Reg. 1308/2013
	II.Quality label information	Type of milk	1.pasteurized<br>2 row	National Code, art.83, general requirements
		% min fat on dry matter		National Code, art.83, general requirements
		% max humidity w/w		National Code, art.83, general requirements
		Production date		National Code, art.83, general requirements

	Packaging date		National Code, art.83, general requirements
	Packaging identification number	serial identification number of product's package	National Code, art.83, general requirements
	Quality label mark	presence of quality mark:<br>1 PDO<br>2 PGI<br>3.other<br>4.absence	National Code, art.83, Traditional cheeses
	Food name as registered	presence (0=no, 1=yes)	National Code, art.83, Traditional cheeses
	Production establishment's address	presence (0=no, 1=yes)	National Code, art.83, Traditional cheeses
	National authority's approval number and mark	presence (0=no, 1=yes)	National Code, art.83, Traditional cheeses
	Production establishment's approval code number	presence<br>(0=no, 1=yes, 2=wrong)	Reg. 854/2004
**III. Nutritional information**	Energy/Energy unit<br>Protein/Protein unit<br>Total fat/Total fat unit<br>Saturated fat/ Saturated fat unit<br>Trans fat/Trans fat unit<br>Carbohydrates/ Carbohydrates unit<br>Sugar/Sugar unit<br>Fibre/Fibre unit<br>Salt/Salt unit<br><br>(insert extra row for each extra nutrient if any)	nutrition declaration table's list of nutrients per 100g or 100ml	Reg. 1169/2011
	Nutrition declaration mandatory particulars	table format or/and nutrients' sequencing compliance<br>(0=no, 1=yes, 2=wrong)	Reg. 1169/2011

Non-mandatory information	IV.Nutritional supplementary information	Portion particulars	presence<br>(0=no, 1=yes, 2=wrong)	Reg. 1169/2011
		Portion size		Reg. 1169/2011
		RI'S particulars	presence<br>(0=no, 1=yes, 2=wrong)	Reg. 1169/2011
		Front of Pack Label schemes(FoPs)	presence (0=no, 1=yes, 2=wrong)	Reg. 1169/2011
		Type of FoP	1. Energy<br>2. Energy +<br>3. other	Reg. 1169/2011
	V.Claims information	Type of claim<br>no1-N	1 nutrition claim<br>2 health claim<br>3 other claim<br>(Any additional type within the above claim types , are described furthermore using the EU Regulation coding system , whisc is also in line with INFORMAS taxonomy)	Reg. 1924/2006<br>-INFORMAS taxonomy
		Wording of claim no1-N	compliance of wording/description of claim using the coding system adapted to EU Regulation<br>(0=no, 1=yes)	Reg. 1924/2006
		Placement of claim<br>no1-N	1 front of pack<br>2 elsewhere on the package (not front of pack)	Reg. 1924/2006
		Format of claim<br>no1-N	1 numerical<br>2 verbal<br>3 symbolic	INFORMAS taxonomy
		Total number of claims for each product	total number of claims for each product and sum of all products	
		Nutrition claims	total number of nutrition claims for each product	
		Health claims	total number of health claims for each product	
		Other claims	total number of 'other' claims for each product	
		Other marks-symbols	type of other claims¨<br>1. origin<br>2. health-related<br>3. sustainability- environmental<br>4. organic<br>5. recycling marks<br>6. natural<br>7. no preservatives<br>8. other	
